# Oral contraceptive use and cancer. Findings in a large cohort study, 1968–2004

**DOI:** 10.1038/sj.bjc.6603260

**Published:** 2006-07-04

**Authors:** M Vessey, R Painter

**Affiliations:** 1 Unit of Health Care Epidemiology, Department of Public Health, University of Oxford, Old Road Campus, Headington, Oxford OX3 7LF, UK

**Keywords:** oral contraceptives, breast cancer, cervical cancer, endometrial cancer, ovarian cancer, cohort study

## Abstract

We examined cancer incidence in relation to oral contraceptive (OC) use in the Oxford Family Planning Association contraceptive study. The study includes 17032 women, recruited at family planning clinics at ages 25–39 years between 1968 and 1974, who were using OCs, a diaphragm, or an intrauterine device. Follow-up data were available until 2004. OC use was not significantly related to nonreproductive cancer. Breast cancer findings (844 cases) likewise were very reassuring (rate ratio (RR) comparing women ever using OCs with those never doing so 1.0, 95% confidence interval (CI) 0.8–1.1). There was a strong positive relationship between cervical cancer incidence (59 cases) and duration of OC use (RR comparing users for 97+ months with nonusers 6.1, 95%CI, 2.5–17.9). Uterine body cancer (77 cases) and ovarian cancer (106 cases) showed strong negative associations with duration of OC use: RRs for 97+ months of use were 0.1 (95%CI, 0.0–0.4) and 0.3 (95%CI, 0.1–0.5) respectively. This apparent protective effect for both cancers persisted more than 20 years after stopping OCs. Combining data for cancers of the cervix, uterine body and ovary, the age adjusted RR for women ever using OCs compared with those never doing so was 0.7 (95%CI, 0.5–0.8). Beneficial effects of OCs on the gynaecological cancers thus outweighed adverse effects.

There has long been concern about oral contraceptive (OC) effects on cancer, especially breast cancer and the reproductive cancers. Many epidemiological studies have been reported which have recently been reviewed by the International Agency for Research on Cancer (IARC). Only a summary of the findings has been published to date (Cogliano *et al*, 2005). It was concluded that (a) there is a small increase in breast cancer risk in current and recent combined OC users which has disappeared 10 years after cessation of use; (b) cervical cancer risk increases with duration of use of combined OCs; (c) hepatocellular cancer risk is increased in long-term users of combined OCs in populations with low frequencies of hepatitis B infection and chronic liver disease; and (d) the risks of endometrial and ovarian cancer are decreased in users of combined OCs, the decrease being greater with longer duration of use. For these cancers, some reduction in risk persists for at least 15 years after cessation of use.

Most epidemiological studies of OCs and cancer have been of case–control design with all the problems this implies (Jick and Vessey, 1978). The Oxford-Family Planning Association (Oxford-FPA) contraceptive study, however, is of cohort design and commenced in the early years of OC use (1968). Findings in the study are, therefore, of special importance in assessing the effects of OCs on cancer risk.

Patterns of cancer incidence in the Oxford-FPA study have been described previously. Our most recent report on breast cancer (Vessey *et al*, 1989) included 189 cases; no evidence was found of an association between OC use and this disease. A report on ovarian cancer (42 cases) and endometrial cancer (15 cases) was published by Vessey and Painter in 1995; an apparent protective effect of OC use was found for both cancers. Findings on cervical cancer were presented by Zondervan *et al* (1996). There were only 33 invasive cases, but the findings strongly suggested that OC use increased risk. Malignant melanoma (32 cases) has also been studied; the reassuring results were reported by Hannaford *et al* (1991).

We present here our latest findings (to the end of 2004) for these and other cancers based on much larger numbers of cases than previously.

## MATERIALS AND METHODS

The methods used in the Oxford-FPA study have been described (Vessey *et al*, 1976). Briefly, 17032 women were recruited at 17 family planning clinics in England and Scotland from 1968 to 1974. At entry, each woman was aged 25–39 years, married, white, British, willing to cooperate and either a current OC user of at least 5 months standing or a current diaphragm or intrauterine device user of at least 5 months standing without previous OC use. At recruitment, each woman was asked about her birth date, child-bearing history, contraceptive history, height, weight, social class (based on her husbands occupation), smoking habits and medical history. The records of each woman were flagged in the National Health Service (NHS) central registries to provide automatic notification of deaths and cancer registrations.

Of the 17 032 women recruited, 41% were from social classes I–II and only 10% were from social classes IV–V. In all, 69% were nonsmokers or ex-smokers; only 14% smoked 15 or more cigarettes daily. Mean body mass index (weight (kg)/height (m)^2^) was only 22.6. These favourable characteristics reflect the fact that family planning clinics attract women who are of higher social class and are more health conscious than the general population.

Where possible, annual follow-up was based on clinic visits. Each woman was asked about pregnancies, changes in contraception, cervical cytology and hospital referrals. Where appropriate, questions were also asked about hormone replacement therapy (HRT) and menopausal status. If necessary, women were sent a postal follow-up form, interviewed by telephone or visited at home. After 5 years, 32% of women were followed by the clinic, 47% by post, 18% by telephone and 2% by home visit. After 10 years, the corresponding figures were 12, 61, 26 and 1%.

A part-time research assistant worked in each clinic. Yearly contact was maintained with participants until age 45 with an annual loss to follow-up for noncooperation or failure to trace of about 0.4%. Cancer diagnoses were confirmed by obtaining hospital summaries. All diagnostic coding was carried out by MV.

At age 45, each woman was allocated to one of three groups: OCs never used, OCs used for 8 years or more, and OCs used for less than 8 years. To reduce costs, only those in the first two groups were subsequently followed-up annually; this continued until mid-1994. Follow-up of those in the remaining group from reaching age 45 until mid-1994 was limited to cancer registrations and deaths notified by the NHS central registries. From mid-1994, information recorded for all the women has been limited to death and cancer notifications from the registries.

The cancers (or cancer groups) considered in this report are listed below. For each cancer, the ICD Code (8th Revision) is given in italics followed by the number of cases. Oesophageal and gastric cancer (*150–151*; 42), cancer of colon and rectum (*153–154*; 131), lung cancer (*162*; 115), malignant melanoma of skin (*172*; 94), other skin cancer (*173*; 352), breast cancer (*174*; 844), cervical cancer (*180*; 59), uterine body cancer (*182*; 77), ovarian cancer (*183.0*; 106), bladder and kidney cancer (*188–189*; 56), lymphomas and leukaemias (*200–208*; 130), other cancers of known site (*140–239* less the above; 150), cancers of uncertain site (*195–199*; 48). None of the uterine cancers was of unknown site while 87% of the uterine body cancers were endometrial in type. In any given analysis, women known to have had the relevant cancer before recruitment were omitted.

The analysis is based on computation of woman-years of observation. Indirectly standardised incidence rates and rate ratios were calculated as described by Vessey *et al* (1976). For significance tests and calculation of 95% confidence intervals (CIs) we used methods given by Breslow and Day (1987). We examined cancer incidence in relation to four categories of total duration of OC use (never used, 1–48 months, 49–96 months, 97 or more months), and to five categories of interval since last OC use (never used, 1–48 months, 49–144 months, 145–240 months, 241 or more months). All analyses took age into account (25–34, 35–39, 40–44, 45–49, 50–54, 55–59, 60 or more). The following variables were also assessed as possible confounders in the OC analyses for each cancer: parity (0, 1, 2, 3, 4 or more births), social class at entry (I–II, III, IV–V and other, Registrar General's Classification), smoking at entry (never smoked, ex-smoker, 1–14 cigarettes daily, 15 or more cigarettes daily) and body mass index at entry (up to 19.9, 20–21.9, 22–23.9, 24–25.9, 26–27.9, 28 or more kg/m^2^). We also examined the influence of age at first term pregnancy (nulliparous, up to 19, 20–24, 25–29, 30 or more) on breast cancer incidence and of age at first marriage (up to 17, 18–19, 20–21, 22 or more) on cervical cancer incidence. Variables having an effect on the age adjusted trend of the cancer concerned at the 10% or higher level of statistical significance were considered to be potential confounders.

Data about hysterectomy, oophorectomy, age at menopause and use of HRT were also judged to be of potential importance. Information about these variables was collected only while women were under annual follow-up. Analyses were undertaken within the relevant part of the database. OC use showed no important association with hysterectomy, oophorectomy or age at menopause. Among those who had never used OCs, 17% used HRT for more than 2 years; the corresponding figure for those who had used OCs for 8 or more years was slightly higher (21%). Viewed overall, we were reassured that our inability to take any of these variables into account in the analyses presented here is unlikely to have distorted the results.

Preparations containing 50 *μ*g of oestrogen made up 67% of OC exposure. The corresponding figure for OCs containing more oestrogen was 2% and for OCs containing less oestrogen was 18%. Progestogen-only OCs made up the remainder (13%). As exposure was dominated by one broad group of OCs and considering the small numbers of cancers in some categories, the data were not analysed by OC formulation.

## RESULTS

### Woman-years of observation

The cancers analysed occurred during 540 000 woman-years of observation. The woman-years were (by chance) distributed more or less equally between the age groups. The group of nonusers of OCs included 187 000 woman-years (35% of the total) while the corresponding figure for the group using OCs for 97 months or more was 116 000 (21% of the total). There were 112 000 woman-years (21% of the total) in the group that had used OCs within the last 48 months while the corresponding figure for the group who had last used the pill 241 or more months before was 73 000 (14% of the total).

### Cancer in relation to duration of OC use

The findings are summarised in Table 1 in ICD code order. Looking first at the nonreproductive cancers, there is no significant evidence of an association between the risk of any of these cancers and duration of OC use. Nonetheless, the rate ratios for lung cancer are all somewhat elevated in OC users; this may reflect residual confounding, including inadequate adjustment for smoking which was slightly more common at entry among OC users than nonusers (Vessey *et al*, 1976). Within the group of other cancers of known site, there were three women with hepatocellular and three with biliary tract cancer. Two of the former and two of the latter were past OC users; all had used OCs for long periods (123, 129, 105 and 177 months, respectively).

Breast cancer incidence was unrelated to duration of OC use; all the rate ratios were close to unity (Table 1). Comparing ever-users with never-users, the rate ratio was 1.0(0.8–1.1). Cervical cancer showed a strong positive association with duration of OC use; the rate ratios increased from 2.9(0.9–9.9) for the up to 48 months group to 6.1(2.5–17.9) for the 97 or more months group. The ever-used to never-used comparison gave a rate ratio of 4.2(1.8–12.0).

Both uterine body and ovarian cancer were strongly negatively associated with OC use; the ever-used to never-used comparisons yielded rate ratios of 0.3(0.2–0.6) and 0.5(0.3–0.7), respectively. While the rate ratios for uterine body cancer declined steadily with increasing duration of use, those for ovarian cancer were below unity only in the last two duration groups, suggesting that a minimum period of use may be necessary to obtain the beneficial effect.

### Cancer in relation to interval since last OC use

Table 2 shows that there is no significant evidence of an association between interval since last OC use and any of the nonreproductive cancers. All four women with liver or biliary duct tumours who had used OCs had discontinued them more than 144 months previously.

The data for breast cancer are once again completely reassuring. Approximately half of the cervical cancers are concentrated in the shortest interval group and this group also has the highest rate ratio (5.2; 2.1–15.5). In an analysis that separated out the group of 21 cancers occurring in current or recent (within 12 months) OC users, the rate ratio was 6.8(2.6–20.5). Nonetheless, significantly increased rate ratios are apparent in the 49–144 months group (3.9; 1.4–12.3) and the 145–240 months group (4.6; 1.5–15.6) suggesting that some adverse effect of OCs on cervical cancer may persist for many years after cessation of use.

By contrast, uterine body and ovarian cancers are concentrated in the long interval groups (Table 2). Rate ratios are below unity in every cell for both cancers although there is a trend towards increasing rate ratios with time elapsed since OCs were stopped for uterine body cancer which just reaches statistical significance. Nonetheless, even in the longest interval group the rate ratios for both cancers are just significantly below unity (0.5, 95% CI 0.3–0.9 and 0.6, 95% CI 0.3–1.0, respectively). These data support the view that the protective effect of OCs against these two cancers is very persistent.

## DISCUSSION

The data collected in the Oxford-FPA study are known to be reliable (Vessey, 1998). Nonetheless, the present analyses have limitations that must be considered before discussing the results.

First, annual follow-up of women who had used OCs for less than 8 years ceased at age 45. Information about the confounding variables considered in the present analysis was, however, available for these women. Furthermore, since pill use was generally complete by age 45 (thus only 3% of the women in whom follow-up ceased at that age were still using OCs), accurate information about OC exposure was also available for this group. Indeed, the only way in which the information available for the women in whom annual follow-up stopped at age 45 differed materially from that available for the other women, concerns cancers occurring between age 45 and the general cessation of annual follow-up in mid-1994. For the former group, cancer information in the relevant interval came only from the NHS central registries. For the latter group, cancer information also came from the annual follow-up. It is thus possible that there might be some under-ascertainment of cancer (especially nonmelanoma skin cancer) in the analyses concerning women using OCs for up to 8 years in comparison with the other groups of women. Table 1, however, provides no indication of such under-ascertainment (even in the other skin cancer group) and it seems reasonable to conclude that the study procedures when women reached age 45 have not significantly biased our findings.

Secondly, annual follow-up ceased generally in mid-1994. Consequently, information about cancer beyond mid-1994 (to the present closure date in December 2004) came only from the registries. This implies some under-ascertainment of the different cancers (especially for the most recent years and for nonmelanoma skin cancer). This, however, should not bias the present OC analyses since under-ascertainment beyond mid-1994 is most unlikely to have been affected by OC use.

Thirdly, information about hysterectomy, oophorectomy, age at menopause and HRT use was obtained only while women were being followed-up annually. We have assumed that the available findings apply more generally to the analyses presented here. This assumption is unlikely to have distorted the results. Fourthly, the numbers of cancers included in many of the comparisons are small and many rate ratios have wide CI. Finally, our findings relate mainly to OCs containing 50 *μ*g of oestrogen, a high dose by today's standards.

The extent to which our findings can be generalised is important. Women attending family planning clinics are of higher social class and more health conscious than women in general. Despite this, we see no reason why our findings should not be broadly applicable, especially in terms rate ratios (which we have chosen to present) rather than incidence rates.

In assessing our findings in relation to other work, it must be stressed that there is a substantial literature on OCs and cancer. In the interests of space, and to obtain an unbiased interpretation of the literature, reference is best made to reliable independent overviews. The IARC produces such overviews as part of their monograph series. Monograph 72 (IARC, 1999) provides an overview of work published to mid-1998. An updated volume, Monograph 91, has recently been completed but has yet to be published. Nonetheless, the main conclusions of the group are available (Cogliano *et al*, 2005) and have been summarised in our introduction.

Our study is too small to provide useful information about hepatocellular cancer save to underline its rarity in the United Kingdom. The negative findings for the other nonreproductive cancers are in line with the IARC conclusions (Cogliano *et al*, 2005). We also found no relationship between OC use and breast cancer, based on substantial numbers of cases (844). Only 192 of these cases, however, occurred within 144 months of cessation of OC use and the rate ratios in the intervals up to 48 months and 49–144 months were 1.1(0.8–1.4) and 0.9(0.7–1.1), respectively. These data do not exclude a small increase in risk (about 20% in current users declining to no increase 10 years after stopping) as described by the Collaborative Group on Hormonal Factors in Breast Cancer (1996) on which the IARC conclusions appear largely to be based.

Our findings, despite the small number of cases (53), provide strong evidence for a considerable increase in cervical cancer risk in OC users. The risk increases with duration of OC use, in line with the IARC report (Cogliano *et al*, 2005). We published broadly similar findings for cervical cancer from the Oxford FPA study 10 years ago (Zondervan *et al*, 1996). This earlier analysis included 33 women with invasive cancer and 280 with cervical intraepithelial neoplasia. It was possible to take several factors into account in that analysis not considered here including use of barrier contraceptives, male and female sterilisation and abortion history. In addition, the (very) limited study data on sexual behavioural factors and the incomplete study information on cytological screening were discussed. We concluded that none of the factors considered was likely to explain the association between OC use and cervical cancer.

Human papilloma virus (HPV) infection in relation to OC use and cervical cancer is also important. Our study provides no information on this topic which is, however, considered in detail in a recent systematic review by Smith *et al* (2003). In that review, the summary relative risks for cervical cancer were 1.1 (1.1–1.2) for less than 5 years OC use, 1.6 (1.4–1.7) for 5–9 years use and 2.2 (1.9–2.4) for 10 or more years use. The figures were similar when the analysis was limited to HPV positive women. These relative risks are much lower than our rate ratios suggesting, perhaps, that some unknown factor is operating in our study to elevate the apparent risk associated with OC use. Further reliable information about OCs and cervical cancer is available in a report from the Collaborative Group on Epidemiological Studies of Cervical Cancer (submitted for publication). This report shows that the elevated risk in OC users has disappeared 10 years after cessation of use. Our findings (Table 2) are at variance with this result: we do not know why.

Our findings for uterine body cancer are again in line with those of the IARC (Cogliano *et al*, 2005). The risk of developing such a cancer decreased markedly with increasing duration of OC use. There was only one case among users who had last taken OCs within the previous 144 months and the results given in Table 2 suggest that some protective effect may continue for more than 20 years after stopping. Our findings fit in well with other published work although we have found a stronger protective effect than has been reported in recent reviews (IARC, 1999; Population Reports, 2000; Deligeoroglou *et al*, 2003).

Our data strongly support the conclusions of the IARC (Cogliano *et al*, 2005) and recent reviews in indicating a protective effect of OCs against ovarian cancer (Population Reports, 2000; Deligeoroglou *et al*, 2003; Riman *et al*, 2004). In our study, there was no effect with durations of OC use up to 48 months; marked protection was, however, apparent in the longer duration of use groups. There was little evidence that the protective effect waned with the passage of time since OCs were stopped; indeed, a protective effect was still apparent in women discontinuing OC use more than 20 years before (Table 2).

Does the beneficial effect of OCs on cancers of the uterine body and ovary outweigh the harmful effect on cervical cancer? To investigate this question, we pooled the data for the three cancers and carried out a simple analysis, adjusting for the effects of age. The rate ratio comparing women who had ever used OCs with those who had never done so was 0.7 (95% CI 0.5–0.8). The adjusted incidence rates per 1000 woman-years were 0.37 (95% CI 0.31–0.45) and 0.57 (95% CI 0.47–0.69) respectively. In our study, these data indicate an overall beneficial effect of OC use on cancer risk.

In summary, our findings on cancer and OC use are clear cut and largely consistent with reviews of other studies. The results are of particular importance because they are derived from a cohort study with reliable data on OC exposure and cancer incidence and considerable quantities of information about long-term OC use (more than 8 years) and about OC effects 20 years and more after OC discontinuation. Furthermore, our study provides information about the full range of cancers and not just about breast cancer and the reproductive cancers. Our results, of course, apply only to the OCs widely used in the 1970s and 1980s; other studies are required to evaluate the long-term effects of the preparations used today. Many older women, however, whose use of OCs ended 20–25 years ago, should be reassured by our findings.

## Figures and Tables

**Table 1:** Cancer (ICD order) in relation to total duration of OC use

	**Total duration of oral contraceptive use (months)**									
	**Nonuser**		**Up to 48**		**49 to 96**		**97 or more**		**All durations**	
**Cancer site**	**No**	**RR**	**No**	**RR**	**No**	**RR**	**No**	**RR**	**No**	**RR**
Oesophagus and stomach (1,2,3)	19	1.0	7	0.8 (0.3–1.9)	9	0.7 (0.3–1.7)	7	0.5 (0.2–1.2)	23	0.6 (0.3–1.3)
Rectum and colon (1,4)	56	1.0	26	1.1 (0.6–1.7)	23	0.8 (0.4–1.2)	26	0.8 (0.5–1.2)	75	0.8 (0.6–1.2)
Lung (1,2,3,5)	30	1.0	17	1.2 (0.6–2.3)	30	1.5 (0.8–2.5)	38	1.4 (0.8–2.3)	85	1.4 (0.9–2.1)
Malignant melanoma (1,2,3,6)	40	1.0	9	0.4 (0.2–0.9)	21	0.9 (0.5–1.5)	24	1.0 (0.6–1.7)	54	0.8 (0.5–1.2)
Other skin (1,2,3)	128	1.0	61	1.1 (0.8–1.5)	86	1.2 (0.9–1.6)	77	1.0 (0.7–1.3)	224	1.1 (0.9–1.4)
*Breast* (1,3,7)	314	1.0	141	0.9 (0.8–1.1)	182	0.9 (0.8–1.1)	207	1.0 (0.8–1.2)	530	1.0 (0.8–1.1)
*Uterine cervix* (1,2,3,5,8)	6	1.0	9	2.9 (0.9–9.9)	16	3.3 (1.2–10.4)	28	6.1 (2.5–17.9)	53	4.2 (1.8–12.0)
*Uterine body* (1,4)	50	1.0	12	0.6 (0.3–1.1)	11	0.4 (0.2–0.8)	4	0.1 (0.0–0.4)	27	0.3 (0.2–0.6)
*Ovary* (1)	58	1.0	28	1.0 (0.6–1.7)	10	0.3 (0.1–0.6)	10	0.3 (0.1–0.5)	48	0.5 (0.3–0.7)
Kidney and bladder (1,4)	24	1.0	6	0.6 (0.2–1.5)	15	1.1 (0.6–2.2)	11	0.7 (0.3–1.5)	32	0.8 (0.5–1.5)
Lymphomas and leukaemias (1)	47	1.0	23	1.1 (0.6–1.8)	28	1.0 (0.6–1.7)	32	1.1 (0.7–1.7)	83	1.1 (0.7–1.6)
Other known (1)	54	1.0	34	1.2 (0.8–1.9)	26	0.8 (0.5–1.3)	36	1.1 (0.7–1.7)	96	1.0 (0.7–1.5)
Uncertain (1,3)	14	1.0	6	1.0 (0.3–2.7)	9	1.1 (0.4–2.6)	19	1.8 (0.9–3.9)	34	1.4 (0.7–2.7)

Numbers within parentheses indicate confounding variables taken into account in each analysis. RR indicates the rate ratio with 95% CI. Nonusers are taken as the reference group.

*Confounding variables*. 1, age; 2, social class; 3, smoking; 4, body mass index; 5, parity; 6, height; 7, age at first-term pregnancy; 8, age at first marriage.

*Trend tests.* Uterine cervix, uterine body, ovary: all *P*<0.001. All other cancers: not significant.

**Table 2:** Cancer (ICD order) in relation to interval since OCs last used

	**Interval since last oral contraceptive use (months)**							
	**Up to 48**		**49–144**		**145–240**		**241 or more**	
**Cancer site**	**No**	**RR**	**No**	**RR**	**No**	**RR**	**No**	**RR**
Oesophagus and stomach	4	0.9 (0.2-2.8)	3	0.5 (0.1-1.6)	8	0.8 (0.3-1.9)	8	0.5 (0.2-1.3)
Rectum and colon	5	0.7 (0.2-1.7)	8	0.6 (0.2-1.3)	25	0.9 (0.6-1.5)	37	0.9 (0.6-1.4)
Lung	2	1.2 (0.1-4.7)	14	1.9 (0.9-3.6)	24	1.2 (0.7-2.1)	45	1.4 (0.8-2.2)
Malignant melanoma	5	0.4 (0.1-0.9)	22	1.4 (0.8-2.4)	11	0.6 (0.3-1.1)	16	0.8 (0.4-1.5)
Other skin	9	0.6 (0.3-1.1)	31	0.9 (0.6-1.3)	75	1.2 (0.9-1.6)	109	1.2 (0.9-1.5)
*Breast*	79	1.1 (0.8-1.4)	113	0.9 (0.7-1.1)	184	1.1 (0.9-1.3)	154	0.9 (0.7-1.1)
*Uterine cervix*	26	5.2 (2.1-15.5)	15	3.9 (1.4-12.3)	10	4.6 (1.5-15.6)	2	1.3 (0.1-7.2)
*Uterine body*	0	—	1	0.1 (0.0-0.5)	8	0.3 (0.1-0.7)	18	0.5 (0.3-0.9)
*Ovary*	1	0.1 (0.0-0.5)	10	0.5 (0.2-0.9)	14	0.5 (0.2-0.9)	23	0.6 (0.3-1.0)
Kidney and bladder	1	0.3 (0.0-1.9)	8	1.1 (0.4-2.6)	7	0.6 (0.2-1.4)	16	0.9 (0.5-1.8)
Lymphomas and leukaemias	11	1.2 (0.6-2.4)	11	0.8 (0.4-1.6)	28	1.3 (0.8-2.1)	33	1.0 (0.6-1.6)
Other known site	19	1.0 (0.6-1.7)	15	0.8 (0.4-1.5)	28	1.1 (0.7-1.6)	34	1.0 (0.6-1.6)
Uncertain site	2	1.9 (0.2-8.2)	4	1.1 (0.3-3.5)	11	1.4 (0.6-3.3)	17	1.4 (0.6-3.0)

Confounding factors taken into account in each analysis are shown in Table 1. RR indicates the rate ratio with 95% CI. Nonusers are taken as the reference group but data are not shown here – see Table 1.

*Trend tests* Uterine body: *P*<0.05. All other cancers: not significant.
